# Sex Differences in Intestinal Microbiota and Their Association with Some Diseases in a Japanese Population Observed by Analysis Using a Large Dataset

**DOI:** 10.3390/biomedicines11020376

**Published:** 2023-01-27

**Authors:** Kouta Hatayama, Kanako Kono, Kana Okuma, Kazumi Hasuko, Hiroaki Masuyama, Yoshimi Benno

**Affiliations:** 1 Symbiosis Solutions Inc., Tokyo 101-0064, Japan; 2 Benno Institute for Gut Microflora, Saitama Industrial Technology Center, Saitama 333-0844, Japan

**Keywords:** intestinal microbiota, sex differences, age, Japanese, disease

## Abstract

In recent years, many studies have focused on the relationship between intestinal microbiota and human health, but the impact of sex has not yet been sufficiently investigated. In this study, sex differences in the intestinal microbiota of a Japanese population were investigated by age group, using a large dataset constructed for a cross-sectional study. α-diversity analysis indicated that the impact of sex differences varied among the 20s–50s age groups but tended to be smaller among the 60s–70s age groups. *Fusobacterium*, *Megamonas*, *Megasphaera*, *Prevotella*, and *Sutterella* were more common among males, whereas *Alistipes*, *Bacteroides*, *Bifidobacterium*, *Odoribacter*, and *Ruthenibacterium* were common among females. Next, intestinal bacteria potentially associated with 12 diseases were investigated for each sex. The results indicate that many of these differ between males and females, and among age groups. Thus, sex and age should be considered for studies on intestinal microbiota and disease association, prevention, and treatment approaches that target them.

## 1. Introduction

The intestinal microbiota of Japanese individuals exhibits a unique composition that differs from that of individuals in other countries [1]. Additionally, intestinal microbiota varies among individuals, even in healthy Japanese individuals. Age is one factor contributing to these differences [2], while sex could be another [3]; however, previous studies have reported conflicting results regarding the impact of sex differences on the intestinal microbiota of Japanese populations [2,4,5,6]. This may have been influenced by the selection of target populations and sample sizes. To comprehensively understand the role of sex differences on the intestinal microbiota, it is necessary to analyze datasets with a large sample size that take age into account.

Recently, it has been reported that intestinal microbiota is associated with the onset and course of various diseases. That is, the intestinal microbiota in patients with various diseases were found to be characteristically different from those of healthy individuals. These diseases include inflammatory bowel disease [7], allergies (such as Japanese cedar pollinosis [8,9]), autoimmune diseases (such as systemic lupus erythematosus and rheumatoid arthritis [10,11]), lifestyle-related diseases (for example, obesity and diabetes [12,13,14]), liver diseases (such as liver cirrhosis and nonalcoholic fatty liver disease [15,16,17,18]), heart diseases (such as atherosclerosis and heart failure [19,20]), cancers (for instance, colorectal cancer [21,22]), and neurological/mental illnesses (such as depression, autism, and Parkinson’s disease [23,24,25]). If sex differences exist in the human intestinal microbiota, disease-associated intestinal bacteria may also differ between males and females.

Therefore, we investigated the differences among intestinal microbiota of the two sexes in a disease-free Japanese population. A large-scale dataset constructed for a cross-sectional study in Japan was used. We then determined whether there were sex differences in intestinal bacteria potentially associated with disease. Twelve diseases were studied, and the data of the affected participants were included in this dataset. The results indicated that sex differences exist in the intestinal microbiota of the Japanese population, that these differences are influenced by age, and that the intestinal bacteria potentially associated with each disease differ between males and females. Therefore, age and sex are crucial factors in studies related to the association, treatment, and prevention of disease-causing intestinal microbiota.

## 2. Materials and Methods

### 2.1. Database, Dataset, and Study Populations

**Figure 1:**
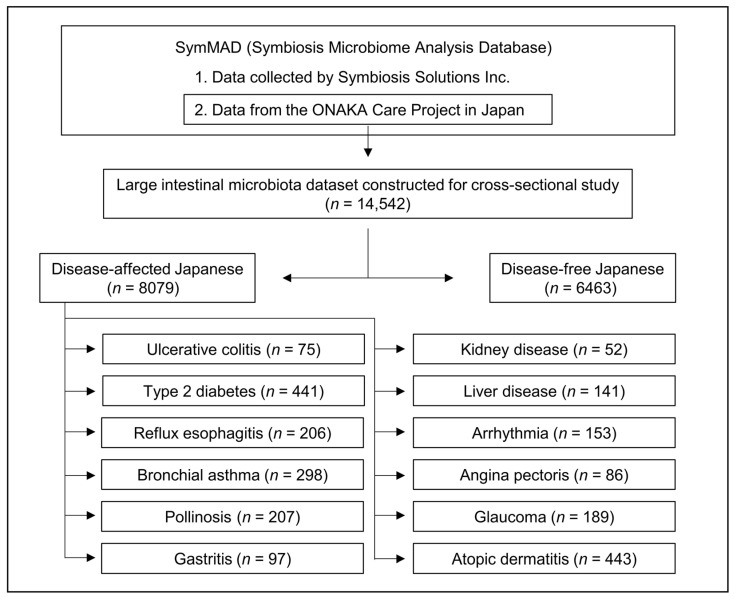
Database, dataset, and study populations.

This study used partial data from SymMAD (Symbiosis Microbiome Analysis Database; data not publicly available for privacy reasons) [26], which is a large Japanese intestinal microbiota database owned by Symbiosis Solutions Inc. (Figure 1). SymMAD consists of two major groups of data. The first consists of data from Symbiosis Solutions Inc., collected with individual participation consent, while the second is composed of data from the ONAKA Care Project, which was designed for a cross-sectional study. The ONAKA Care Project (September 2011–March 2021) was designed and managed by the Benno Laboratory, with the RIKEN Baton Zone Program, RIKEN Cluster for Science, Technology, and Innovation Hub (Saitama, Japan). Japanese volunteers aged 20–79 years were recruited through seminars on intestinal microbiota held in various regions of Japan and through announcements in Japanese newspapers. The data is composed of intestinal bacterial DNA extracted from stool samples of Japanese people collected by the Benno Laboratory and analyzed by the Japan Agricultural Frontier Development Organization (Tokyo, Japan). This second group data is licensed to Symbiosis Solutions Inc. We used only the dataset generated from the second group in SymMAD data (i.e., the data from the ONAKA Care Project). This study involved 14,542 participants for whom intestinal microflora and questionnaire data were obtained. Those who met the following criteria were excluded from the study: participants with insufficient data for intestinal microbiota, currently pregnant or nursing, taking antibiotics, who were non-Japanese, or who passed stools that were occult blood-positive or required enema. 

From the Japanese intestinal microbiota dataset constructed for the cross-sectional study, we selected a disease-free population as well as those with 12 diseases (ulcerative colitis, type 2 diabetes, gastritis, reflux esophagitis, kidney disease, liver disease, arrhythmia, angina pectoris, glaucoma, bronchial asthma, pollinosis, and atopic dermatitis) based on the questionnaire data (Figure 1).

### 2.2. Ethical Considerations

This study was part of the ONAKA Care Project, which was approved by the Research Ethics Committee of RIKEN (approval number: Wako 3 27-22 and approval Date: 1 April 2016). Written informed consent was obtained from all participants.

### 2.3. Questionnaire Survey

Background information such as age, sex, and health status (including disease status), was collected through the participants’ self-reports using a questionnaire in parallel with stool collection. Regarding disease status, the participants notified whether they had been diagnosed by a physician.

### 2.4. Collection of Stool Samples

Stool samples were collected by the participants using a stool collection kit (Techno-Suruga Laboratory Co., Ltd., Shizuoka, Japan). The samples were then suspended in guanidine thiocyanate (GTC) solution (100 mM Tris-HCL (pH 9.0), 40 mM Tris-EDTA (pH 8.0), 4 M guanidine thiocyanate, and 0.001% bromothymol blue and then mailed to the Benno Laboratory without temperature control.

### 2.5. DNA Extraction

DNA was extracted from the stool samples using the following process: 400 mg glass beads and 600 µL phenol–chloroform solution (1:1 tris-EDTA saturated phenol and chloroform mixture) were added to stool samples suspended in 1 mL GTC solution, along with 100 µL 10% SDS. The samples were disrupted at 7,000 rpm for 20 s using a bead crusher (MagNA Lyser; Roche Molecular Diagnostic, Mannheim, Germany) and incubated at 70 °C for 10 min. This process was repeated twice. The samples were immediately cooled in a water bath and centrifuged at 10,621× *g* for 5 min, then 700 µL of the supernatant was transferred to a 1.5 mL tube. Approximately 700 µL of cooled isopropanol and 70 µL of a 3 M sodium acetate solution were added to the supernatant and mixed by inversion. Each sample was centrifuged at 20,800× *g* for 5 min; the supernatant was discarded, and the DNA pellets obtained were washed twice with 70% ethanol. The pellets were dissolved in 200 µL of TE buffer and stored at −80 °C until DNA sequencing analysis.

### 2.6. DNA Sequencing

The variable regions V1–V3 of the 16S rRNA gene were sequenced using 35F and 520R primers [26,27]. DNA sequencing using the MiSeq system (Illumina, San Diego, CA, USA) was performed according to the method described by Kono et al. [26], with the exception of Q5 High-Fidelity DNA Polymerase (New England Biolabs, Ipswich, MA, USA) being used for PCR. 

### 2.7. 16S rRNA Data Analysis

16S rRNA data analysis was performed according to the method described by Kono et al. [26]. The generated overlapping and paired-end fastq files were processed using the DADA2 v.1.16.0 package in R software v. 4.0.3 (R Foundation for Statistical Computing, Vienna, Austria) to create amplicon sequence variants (ASVs) [28]. The filterAndTrim function arguments of DADA2 were truncLen = c(0, 246), maxN = 0, maxEE = 5, truncQ = 4, rm.phix = TRUE, compress = TRUE, multithread = TRUE, verbose = TRUE, and minLen = 50. Using the vegan package (v. 2.5–7) in R, a rarefaction curve was generated for each sample based on the number of reads for each unique ASV. For each rarefaction curve, the minimum number of unique ASVs with a slope of 0.002259329 or less were examined, and the number of sequences plus one was used as the sampling depth for rarefying. 

### 2.8. Intestinal Microbiota Analysis

The α-diversity metrics, including Shannon, Simpson, and Pielou diversity indices, were calculated at the genus level using the vegan package v. 2.5.7 in R v. 4.1.0. 

Non-metric multidimensional scaling (NMDS) based on the Bray–Curtis index was used to visualize β-diversity. For NMDS, the metaMDS function in R v. 4.1.0 vegan v. 2.5.7 package was used. PERMANOVA was performed using the vegan adonis function v. 2.5.7 with permutations = 9999.

The R package ALDEx2 v. 1.26.0 was used to compare intestinal microbiota at genus level between the two groups as previously described by Kono et al. [26]. The microbiota abundance count data were converted into a concentric logarithmic ratio (clr conversion) for this comparison. The Wilcoxon rank-sum test was performed, and correction for multiple testing was conducted using the Benjamini–Hochberg false discovery rate (FDR). Statistical significance was set at *p* < 0.05.

### 2.9. Statistical Analysis

The Wilcoxon rank-sum test was used for between-group data comparisons. The Wilcoxon rank-sum test was performed using the Wilcoxon test function (paired = FALSE, correct = FALSE) in R v. 4.1.0. Statistical significance was set at *p* < 0.05.

## 3. Results

### 3.1. Sex Differences in the Intestinal Microbiota of a Disease-Free Japanese Population

A disease-free population (2136 males and 4327 females) was selected from a Japanese intestinal microbiota dataset constructed for the cross-sectional study (Figure 1), and sex differences in the microbiota were investigated. Composition of the microbiota has been reported to change with age [2]. Therefore, we compared the males and females by age group to suppress this effect. There were no significant age differences between the sexes in any group (Table 1).

**Figure 2:**
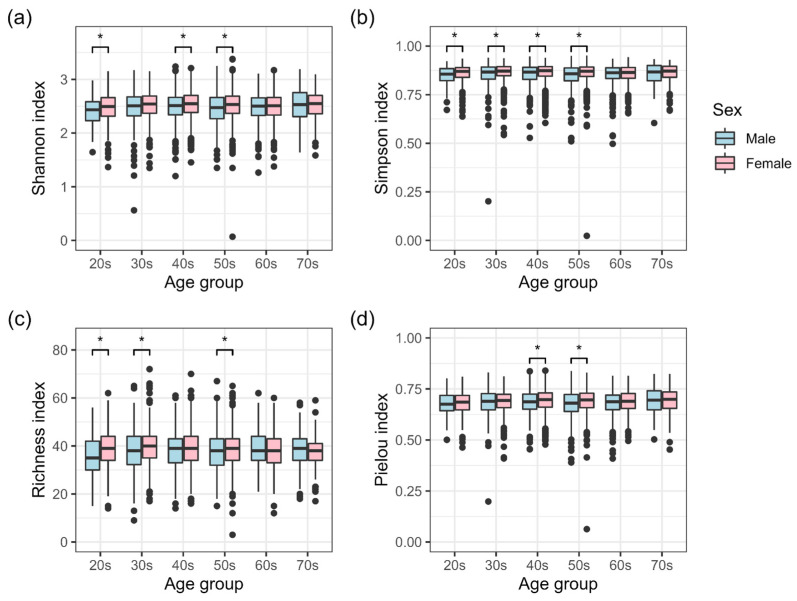
α-diversity of intestinal microbiota in disease-free Japanese males and females. α-diversity indices of Shannon (**a**), Simpson (**b**), Richness (number of genera) (**c**), and Pielou (**d**) are shown for each age group. Asterisks indicate significant differences (*p* < 0.05, Wilcoxon rank-sum test).

The results of intestinal microbiota α-diversity analysis of males and females in each age group are shown in Figure 2. Four indices were used in the α-diversity analysis: Shannon, Simpson, Richness (number of genera), and Pielou at the genus level. Among participants in their 20s, significant differences were found in the Shannon, Simpson, and Richness indices, with females having higher indices than males. Females had significantly higher scores than males on the Simpson and Richness indices in their 30s, on the Shannon, Simpson, and Pielou indices in their 40s, and on all indices in their 50s. Such differences in indices based on age groups (20s–50s) indicated that the impact of sex differences on intestinal microbiota α-diversity varies with age. In contrast, no significant differences were observed in any of the α-diversity indices between males and females in their 60s and 70s.

**Figure 3:**
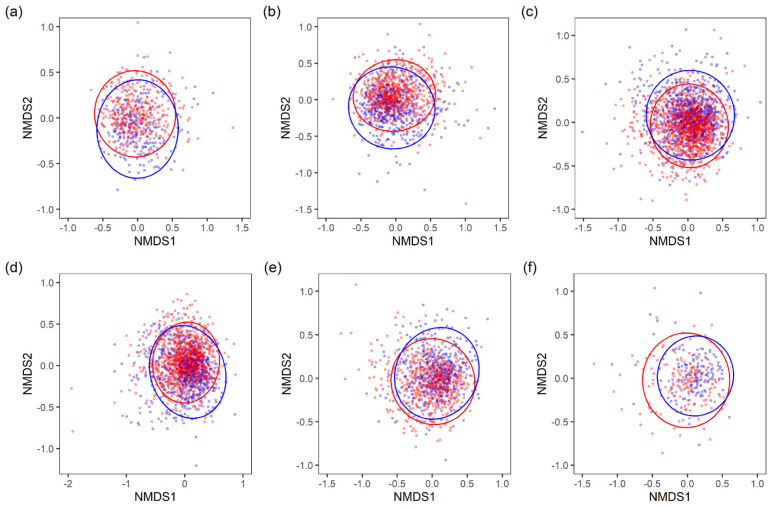
Non-metric multidimensional scaling (NMDS) plotting based on the Bray-Curtis index of disease-free Japanese male and female intestinal microbiota. (**a**) NMDS plots of 20s (Stress = 0.25; PERMANOVA: *p* = 0.0001); (**b**) 30s (Stress = 0.24; PERMANOVA: *p* = 0.0001); (**c**) 40s (Stress = 0.24; PERMANOVA: *p* = 0.0001); (**d**) 50s (Stress = 0.24; PERMANOVA: *p* = 0.0001); (**e**) 60s (Stress = 0.25; PERMANOVA: *p* = 0.0001); (**f**) 70s (Stress = 0.24; PERMANOVA: *p* = 0.0065). Ellipses represent 95% confidence intervals around the centroid. Blue and red in the NMDS plots indicate male and female samples, respectively.

β-diversity was visualized using a non-metric multidimensional scaling (NMDS) plot based on the Bray–Curtis index (Figure 3). The NMDS results for each age group indicated that the composition of the intestinal microbiota was different between males and females (all PERMANOVA P-values between males and females in each age group were significantly different with *p* < 0.05). While there were no significant differences in intestinal microbiota α-diversity between males and females in their 60s and 70s, β-diversity analysis indicated the contrary.

**Figure 4:**
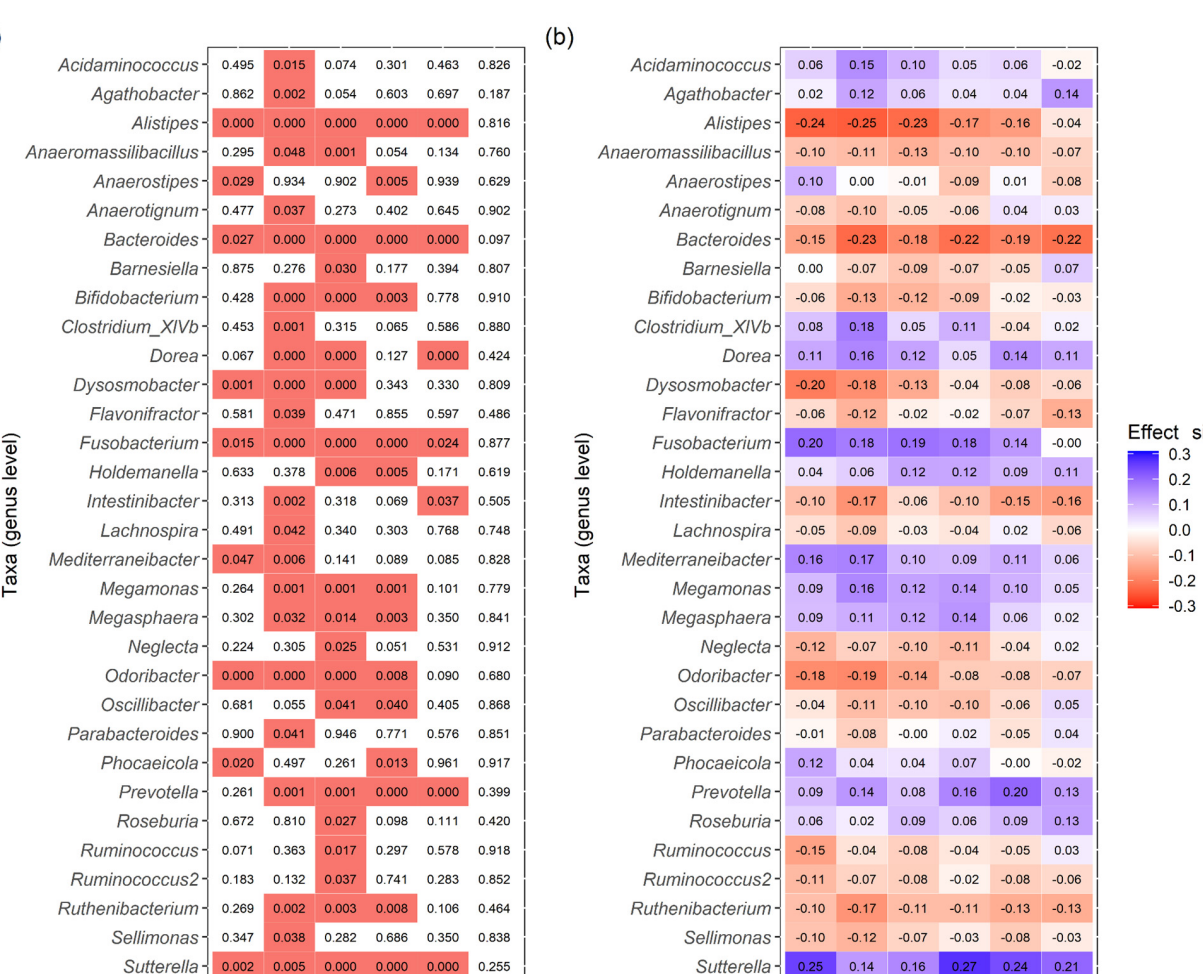
Results of ALDEx2 analysis comparing the intestinal microbiota of disease-free Japanese males and females at the genus level by age groups. (**a**) The FDR- adjusted *p*-values for each taxon are shown. FDR- adjusted *p*-values below 0.05 (significant differences) are indicated in red. A value of 0.000 means below 0.0005. (**b**) The effect size values for each taxon are shown. Positive values of effect size indicate higher abundance of the taxa in males, while negative values indicate higher abundance in females.

To identify intestinal bacteria that were differentially abundant between males and females, the ANOVA-Like Differential Expression tool (ALDEx2) was used. Figure 4 shows the taxa (genus level) for which there were significant differences in CLR-transformed abundance values between males and females in any age group (Wilcoxon rank-sum test, statistically significant: a false discovery rate (FDR)-adjusted *p*-value < 0.05). The number of taxa that showed significant differences was distributed as follows: nine taxa in the 20s age group, 23 in the 30s, 20 in the 40s, 14 in the 50s, seven in the 60s, and zero in the 70s (Figure 4a). The number of bacterial taxa with significant differences was greatest in the 30s, which then decreased with age. In the 30s to 50s age group, *Fusobacterium*, *Megamonas*, *Megasphaera*, *Prevotella*, and *Sutterella* tended to be more common in males, whereas *Alistipes*, *Bacteroides*, *Bifidobacterium*, *Odoribacter*, and *Ruthenibacterium* were more common in females (Figure 4b).

In summary, the results of β-diversity analysis indicated that there are sex differences in the intestinal microbiota composition of a disease-free Japanese population among all age groups, especially the 20s–70s age groups (Figure 3). Analyses of α-diversity and ALDEx2 indicated that the characteristics of sex differences varied specifically in the 20s–50s age groups (Figure 2 and Figure 4). In contrast, no sex differences were observed in the 60s and 70s age groups in terms of α-diversity (Figure 2). ALDEx2 analysis showed that sex differences tended to decrease with age (Figure 4). In other words, these results suggest that sex differences in the intestinal microbiota of a disease-free Japanese population differ in characteristics and extent of difference by age group.

### 3.2. Differences in Intestinal Bacteria Potentially Associated with Disease between Japanese Males and Females

The next step in this study was to investigate whether potential disease-associated intestinal bacteria are different between Japanese males and females. The Japanese intestinal microbiota dataset constructed for the cross-sectional study also contained data on the intestinal microbiota of disease-affected participants (Figure 1). For diseases that affected both males and females across several age groups, intestinal bacteria potentially associated with the diseases were investigated according to age group and sex. Table 2 shows the diseases included in this study and the number of affected participants.

**Figure 5:**
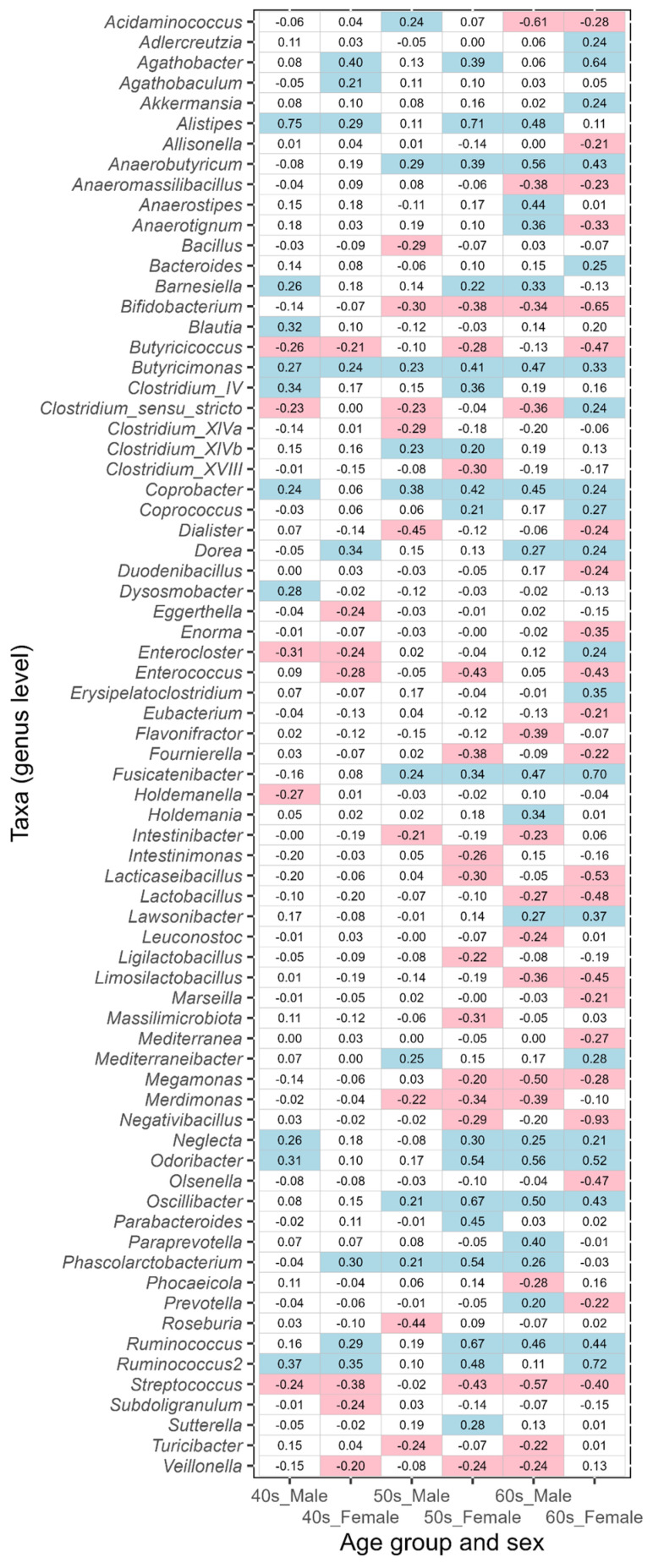
The values of ALDEx2 effect size obtained by comparing the ulcerative colitis disease-affected and disease-free Japanese control groups. The comparisons were performed considering age and sex. Negative values of effect size indicate taxa that are more abundant in the disease-affected group than in the control group, and positive values indicate taxa that are less abundant. The values of effect size less than −0.2 are indicated with pink, and the values greater than 0.2 are indicated with light blue.

**Figure 6:**
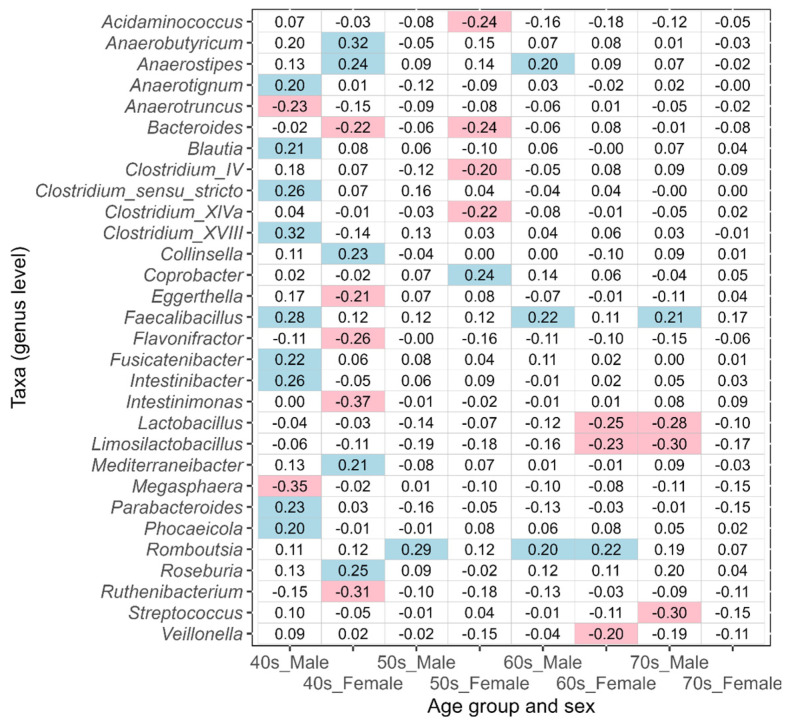
The values of ALDEx2 effect size obtained by comparing the type 2 diabetes disease-affected and disease-free Japanese control groups. The comparisons were performed considering age and sex. Negative values of effect size indicate taxa that are more abundant in the disease-affected group than in the control group, and positive values indicate taxa that are less abundant. The values of effect size less than −0.2 are indicated with pink, and the values greater than 0.2 are indicated with light blue.

**Figure 7:**
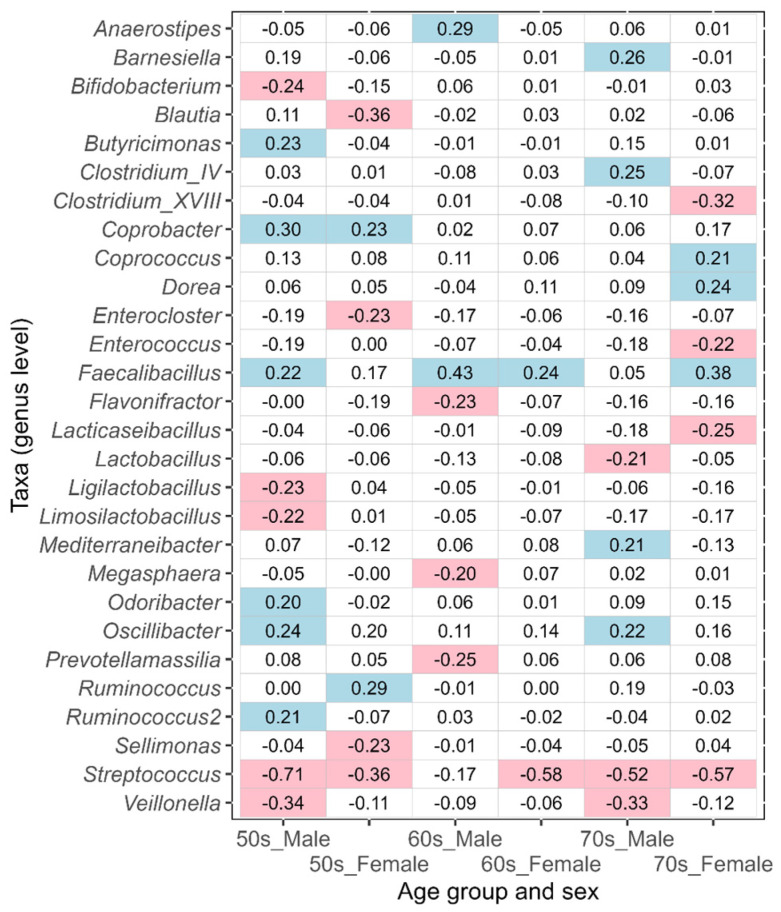
The values of ALDEx2 effect size obtained by comparing the reflux esophagitis disease-affected and disease-free Japanese control groups. The comparisons were performed considering age and sex. Negative values of effect size indicate taxa that are more abundant in the disease-affected group than in the control group, and positive values indicate taxa that are less abundant. The values of effect size less than −0.2 are indicated with pink, and the values greater than 0.2 are indicated with light blue.

**Figure 8:**
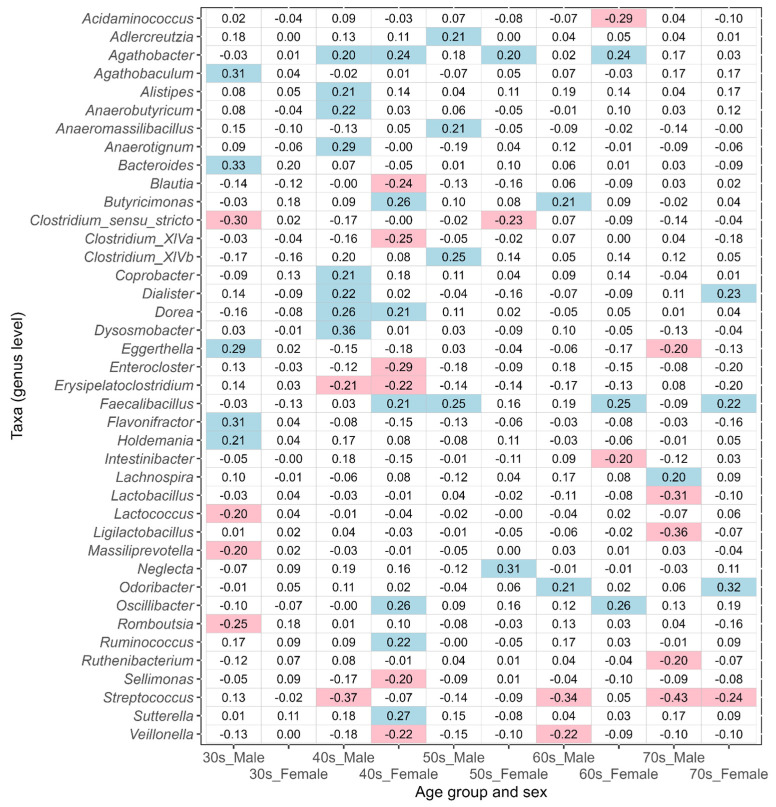
The values of ALDEx2 effect size obtained by comparing the bronchial asthma disease-affected and disease-free Japanese control groups. The comparisons were performed considering age and sex. Negative values of effect size indicate taxa that are more abundant in the disease-affected group than in the control group, and positive values indicate taxa that are less abundant. The values of effect size less than −0.2 are indicated with pink, and the values greater than 0.2 are indicated with light blue.

**Figure 9:**
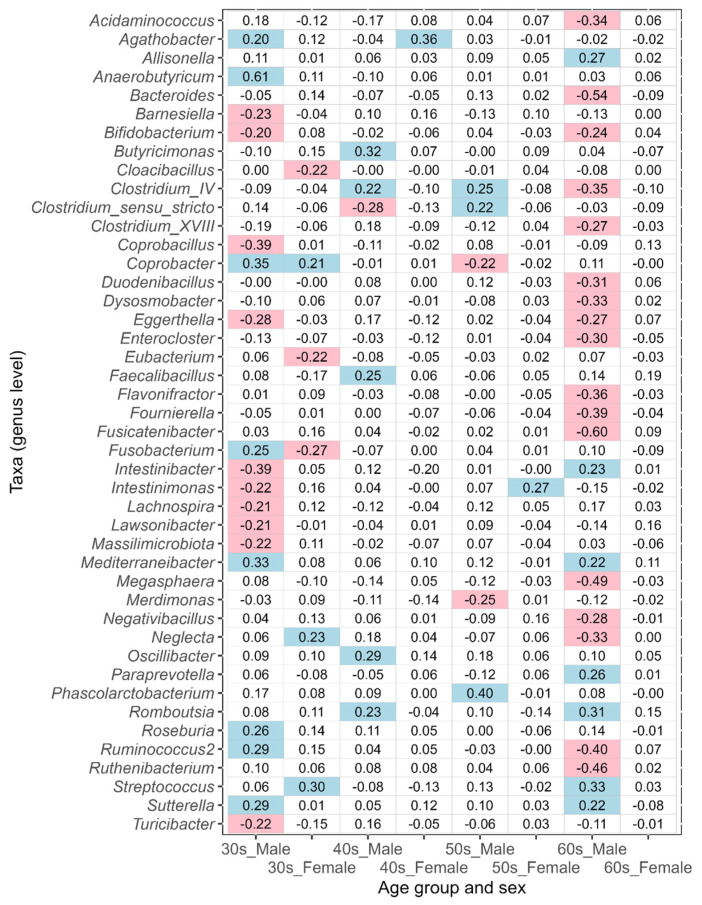
The values of ALDEx2 effect size obtained by comparing the pollinosis disease-affected and disease-free Japanese control groups. The comparisons were performed considering age and sex. Negative values of effect size indicate taxa that are more abundant in the disease-affected group than in the control group, and positive values indicate taxa that are less abundant. The values of effect size less than −0.2 are indicated with pink, and the values greater than 0.2 are indicated with light blue.

In this study, the taxa that showed an absolute value of ALDEx2 effect size greater than 0.2 between the corresponding disease-affected and control (disease-free Japanese, Table 1) groups were defined as the taxa of intestinal bacteria potentially associated with disease. An effect size value of less than −0.2 indicates a taxon that is more abundant in each disease-affected group than in the control group, while a value greater than 0.2 indicates a taxon that is less abundant. The results of ulcerative colitis, type 2 diabetes, reflux esophagitis, bronchial asthma, and pollinosis are shown in Figure 5, Figure 6, Figure 7, Figure 8, and Figure 9, respectively. The results for gastritis, kidney disease, liver disease, arrhythmia, angina pectoris, glaucoma, and atopic dermatitis are shown in Supplementary Figures S1–S7, respectively.

Taxa potentially associated with ulcerative colitis were identified in the 40s–60s age groups. Many of the taxa possibly linked with the disease differed between males and females (Figure 5); however, *Butyricimonas* alone was common between both sexes. Similarly, some other commonalities in taxa were observed, but they did not tend to coincide across age groups (e.g., *Acidaminococcus*).

Many of the taxa potentially associated with type 2 diabetes differed between males and females in the 40s–70s age groups (Figure 6). The only taxon that included both sexes and was potentially associated with the disease was *Romboutsia* in the 60s age group.

In reflux esophagitis, many of the taxa potentially associated with this disease differed between males and females in the 50s–70s age groups (Figure 7). Several commonalities in taxa between the two sexes were observed, but they did not tend to coincide across age groups. As a taxon associated with reflux esophagitis, *Streptococcus* was characteristically observed in males and females of all age groups, except for males in their 60s.

In the 30s–70s age groups, many of the taxa potentially associated with bronchial asthma differed between males and females (Figure 8). Although, several commonalities in taxa between both sexes in the 40s and 70s were observed (e.g., *Agathobacter* in the 40s and *Streptococcus* in the 70s), there were none across the various age groups. 

In pollinosis, many of the taxa potentially associated with this disease differed between males and females in the 30s–60s age groups (Figure 9). The only taxon that included both sexes was *Coprobacter* in the 30s age group.

Many of the taxa potentially associated with gastritis, kidney disease, liver disease, arrhythmia, angina pectoris, glaucoma, and atopic dermatitis differed between males and females in each age group (Supplementary Figures S1–S7, respectively). Some commonalities in taxa between both sexes were observed, but not across age groups.

## 4. Discussion

In the present study, sex differences in intestinal microbiota in a disease-free Japanese population were investigated using a large Japanese intestinal microbiota dataset constructed for a cross-sectional study. The results of α-diversity, β-diversity, and ALDEx2 analyses indicated sex differences in the intestinal microbiota of the population with different characteristics depending on age. Considering that the composition of the intestinal microbiota in the Japanese population changes with age [2], our results are reasonable.

Analysis using ALDEx2 showed bacterial taxa (genus level) with significant differences in CLR-transformed abundance values between disease-free Japanese males and females in the 20s–70s age groups (Figure 4). The number of taxa with significant differences was the highest among those in their 30s, which decreased with increasing age. No taxa with significant differences were found in the 70s age group. In other words, taxa that differed in abundance values between males and females tended to become less common with an increase in age. Focusing on ALDEx2 effect size values, the values of taxa such as *Alistipes* and *Fusobacterium* decreased with increasing age (Figure 4b). In contrast, effect size values of taxa such as *Bacteroides* and *Sutterella* did not change significantly. These results indicate that there may be two trends for the differential abundance of intestinal bacteria: a decreasing trend between males and females as the host ages and a stable one that is maintained regardless of aging.

Analysis using ALDEx2 showed that *Fusobacterium*, *Megamonas*, *Megasphaera*, *Prevotella*, and *Sutterella* tended to be more common in males, especially those in their 30s–50s, whereas *Alistipes*, *Bacteroides*, *Bifidobacterium*, *Odoribacter*, and *Ruthenibacterium* were more common in females (Figure 4). Some of the results indicating the above were consistent with the results of a previous study on Japanese males and females [6]. The study showed significantly higher levels of *Prevotella*, *Megamonas*, *Fusobacterium*, and *Megasphaera* in Japanese males and of *Bifidobacterium*, *Ruminococcus*, and *Akkermansia* in Japanese females [6]. Since there was no significant discrepancy between the results of the previous study and this study, it is valid to assume that our results successfully captured the characteristics of sex differences in the intestinal microbiota of the Japanese population.

Significant differences in α-diversity between males and females in their 20s were observed for the Shannon, Simpson, and Richness indices but not for the Pielou index (Figure 2). These results indicate that sex differences in α-diversity for individuals in their 20s tended to differ more in terms of richness than in terms of evenness. The diversity of the intestinal microbiota in the Japanese population increases substantially after weaning and continues to increase sequentially until the individuals are in their 20s [2]. Taking this into consideration, our results suggest that the number of bacterial genera comprising the intestinal microbiota may increase differently in males and females who are in their 20s. During puberty, hormones act as one of the factors that may contribute to the difference in the number of genera between males and females. A study in mice reported that sex differences in intestinal microbiota are formed under the effect of sex hormones during puberty [29]. Differences in health awareness may be another contributing factor. A study of Japanese males and females (college students aged 19-24) reported that a higher percentage of females were aware of the importance of health [30].

Significant differences in the Shannon index were observed between males and females in their 20s but not between those in their 30s (Figure 2), who showed significant differences in the Simpson index instead. The Shannon index emphasizes the richness component of diversity and rare cover types, whereas the Simpson index highlights the evenness component and dominant cover types [31]. Based on this, it is conceivable that sex differences for individuals in their 30s are smaller with respect to the richness of rarer genera, which are of low relative abundance. For those in their 40s and 50s, there were significant differences between males and females in the Pielou index of evenness, which differed from the results for those in their 20s and 30s. Significant differences were observed only in the 50s age group between the two sexes in all four α-diversity indices. The social and family roles as well as lifestyles of both sexes change with age. In addition to the physical changes due to aging, these changes may influence sex differences in the intestinal microbiota in each age group.

In the 60s and 70s age groups, β-diversity results indicated the presence of sex differences in the intestinal microbiota, but α-diversity indices showed no significant differences (Figure 2 and Figure 3). Sex differences in the intestinal microbiota tended to be smaller in these age groups. One factor may be the decline in estrogen production associated with menopause in females. Estrogen has been reported to be associated with intestinal microbiota [32,33]. With respect to the elderly over 70 years of age, it is possible that the composition of the intestinal microbiota is affected by changes in the intestinal physiology [3,34,35]. It is also possible that the analysis was influenced by limiting the investigation to only disease-free participants. In general, the prevalence of disease increases while the survival rate decreases with older age. Therefore, some intestinal microbiota that are less susceptible to disease and more favorable for survival may be selected.

Among disease-free Japanese participants in their 20s to 50s, the α-diversity of the female intestinal microbiota tended to be higher than that of male participants (Figure 2). Similar results have been reported in studies conducted in regions other than Japan as well [3,36]. This difference has been reported to be more pronounced in young adults than in middle-aged individuals [36], as was the case in this study. Therefore, a higher intestinal microbiota α-diversity in females and the age-dependent changes in the characteristics of sex differences may not be limited to the Japanese population.

Previous studies have reported conflicting results regarding the effect of sex differences on the intestinal microbiota of Japanese populations [2,4,5,6]. This may have been influenced by age-dependent differences. In a population with a mixture of different age groups, it may be difficult to observe sex differences in the intestinal microbiota because of the dilution or offsetting of their differences in each age group. In addition, sex differences in the intestinal microbiota may also be difficult to observe if older adults in their 60s and 70s constitute a large portion of the study population, where the sex differences are small. In fact, studies that reported no sex differences [2] or no strong correlations [4] attempted to observe populations with a mixture of different age groups.

Using a large Japanese intestinal microbiota dataset constructed for a cross-sectional study, this study also investigated the differences in intestinal bacteria potentially associated with disease between Japanese males and females. The results indicated that intestinal bacteria potentially associated with ulcerative colitis, type 2 diabetes, gastritis, reflux esophagitis, kidney disease, liver disease, arrhythmia, angina pectoris, glaucoma, bronchial asthma, pollinosis, and atopic dermatitis differed between the two sexes and among age groups. This phenomenon might be attributed to the underlying sex differences in the intestinal microbiota of Japanese individuals.

However, this study did not aim to elucidate the intestinal bacteria that cause each disease. Therefore, we did not discuss their relationship in depth. It is worth noting, however, that the genera of intestinal bacteria potentially associated with each disease differed between Japanese males and females and that they also differed by age. Although there have been many previous studies on the relationship between disease and intestinal bacteria, there are often discrepancies among them. One possible reason for this may be the differences in sex and age composition of the study populations.

If sex and age are not considered in studies on intestinal microbiota, the results related to them may be overlooked. Approaches for the prevention or treatment of diseases related to intestinal microbiota developed based on such studies may not be sufficiently effective for some people. Therefore, it is extremely important to consider these two factors in future studies on intestinal microbiota and associated diseases as well as treatment approaches developed to target them.

The Japanese intestinal microbiota dataset used in this study consisted of data from participants aged 20–79 years. Therefore, this study did not investigate sex differences in Japanese individuals under and over the age range. To further understand the sex differences in the Japanese intestinal microbiota, future studies should be performed on participants who fall into these age groups.

This study sought to gain a comprehensive understanding of sex differences in intestinal microbiota using the Japanese cross-sectional study dataset. However, in addition to sex and age, there are many other factors that can affect the intestinal microbiota, such as diet, exercise, body mass index, region of residence, smoking, and alcohol consumption. It was difficult to control for these factors in this study. To clarify sex differences in detail, future large-scale studies with controls for various confounding factors are needed.

The dataset used in this study was not specifically designed to investigate sex differences in the intestinal microbiota. All available data on disease-free Japanese males and females from this dataset were selected and used to analyze their sex differences. As a result, the sample sizes were able to be increased, but we were unable to equalize the number of males and females composing the disease-free Japanese population in our dataset. One limitation of this study is that we cannot completely rule out the possibility that differences in sample size between groups of males and females in each age group may have affected our results. To avoid this research limitation in future studies detailing sex differences in intestinal microbiota, participants should be recruited to match the sample sizes of the male and female groups.

The present study shows that intestinal bacteria are potentially associated with certain diseases. However, further studies are needed to elucidate the details of their relationship, specifically taking sex and age into account as demonstrated in this study.

## 5. Conclusions

Although many researchers have been interested in the relationship between intestinal microbiota and disease, few studies have examined sex differences [3]. In this study, we analyzed a large Japanese intestinal microbiota dataset constructed for a cross-sectional study and showed that sex differences exist in the intestinal microbiota and that they are affected by age. Our results contribute to a better understanding of this phenomenon. In addition, the intestinal bacteria associated with disease may also differ by sex and age, indicating the importance of considering these factors when studying their relationship or developing approaches for disease prevention and treatment.

## Figures and Tables

**Table 1:** Number and age of disease-free Japanese males and females in each age group.

Age Group (Age)	Male		Female		*p*-Value ^1^
	n	Age (Mean ± SD)	n	Age (Mean ± SD)	
20s (20–29)	194	24.8 ± 3.0	377	24.6 ± 3.0	0.626
30s (30–39)	382	35.3 ± 2.8	741	35.1 ± 2.9	0.315
40s (40–49)	569	44.5 ± 2.8	1298	44.7 ± 2.8	0.073
50s (50–59)	505	54.2 ± 2.9	1117	53.9 ± 2.8	0.062
60s (60–69)	360	64.2 ± 2.9	625	64.1 ± 2.8	0.713
70s (70–79)	126	72.8 ± 2.7	169	72.8 ± 2.8	0.975

^1^ Wilcoxon rank-sum test for age.

**Table 2:** Diseases included in the study with number of affected participants.

Disease	Sex	Number of Disease-Affected Participants by Age Groups					
		20s	30s	40s	50s	60s	70s
Ulcerative colitis	Male	-	-	9	13	10	-
	Female	-	-	23	12	8	-
Type 2 diabetes	Male	-	-	17	49	120	84
	Female	-	-	17	29	65	60
Reflux esophagitis	Male	-	-	-	19	33	27
	Female	-	-	-	41	49	37
Bronchial asthma	Male	-	11	17	22	27	24
	Female	-	32	50	39	44	32
Pollinosis	Male	-	8	17	10	8	-
	Female	-	17	60	49	38	-
Gastritis	Male	-	-	-	9	15	12
	Female	-	-	-	18	20	23
Kidney disease	Male	-	-	-	8	14	-
	Female	-	-	-	16	14	-
Liver disease	Male	-	-	8	23	16	10
	Female	-	-	21	23	20	20
Arrhythmia	Male	-	-	-	12	36	43
	Female	-	-	-	10	30	22
Angina pectoris	Male	-	-	-	-	26	25
	Female	-	-	-	-	15	20
Glaucoma	Male	-	-	-	24	39	33
	Female	-	-	-	40	33	20
Atopic dermatitis	Male	13	35	41	17	9	11
	Female	46	87	91	62	17	14

-, not applicable.

## Data Availability

The data presented in this study are available upon request from the corresponding author. The data are not publicly available because of privacy concerns.

## Supplementary Materials

The following supporting information can be downloaded at https://www.mdpi.com/article/10.3390/biomedicines11020376/s1, Figure S1: The values of ALDEx2 effect size obtained by comparing the gastritis disease-affected and disease-free Japanese control groups; Figure S2: The values of ALDEx2 effect size obtained by comparing the kidney disease-affected and disease-free Japanese control groups; Figure S3: The values of ALDEx2 effect size obtained by comparing the liver disease-affected and disease-free Japanese control groups; Figure S4: The values of ALDEx2 effect size obtained by comparing the arrhythmia disease-affected and disease-free Japanese control groups; Figure S5: The values of ALDEx2 effect size obtained by comparing the angina pectoris disease-affected and disease-free Japanese control groups; Figure S6: The values of ALDEx2 effect size obtained by comparing the glaucoma disease-affected and disease-free Japanese control groups; Figure S7: The values of ALDEx2 effect size obtained by comparing the atopic dermatitis disease-affected and disease-free Japanese control groups.
