# Supplementary material for: Sex Differences in Intestinal Microbiota and Their Association with Some Diseases in a Japanese Population Observed by Analysis Using a Large Dataset
Source: Biomedicines. 2023 Jan 27;11(2):376. doi: 10.3390/biomedicines11020376 (PMC9953495; doi:10.3390/biomedicines11020376)
Supplement: Supplementary file 1 [file biomedicines-11-00376-s001.zip › biomedicines-2155934-supplementary.pdf]

Taxa (genus level)	50s_Male	50s_Female	60s_Male	60s_Female	70s_Male	70s_Female
Acidaminococcus	-0.30	-0.20	0.09	-0.04	-0.08	-0.10
Agathobacter	0.27	0.16	0.49	0.30	0.07	0.08
Agathobaculum	0.09	-0.01	-0.06	-0.17	0.21	0.08
Akkermansia	-0.07	0.11	0.07	-0.31	0.16	0.01
Alistipes	-0.20	0.04	0.17	-0.06	0.16	0.00
Anaeromassilibacillus	-0.27	0.09	-0.10	-0.01	0.01	-0.10
Bacteroides	0.02	0.33	0.26	-0.33	-0.03	-0.04
Bifidobacterium	-0.02	-0.21	-0.07	0.09	0.05	-0.07
Butyricicoccus	-0.02	0.10	0.04	-0.02	-0.23	0.12
Butyricimonas	-0.39	-0.02	0.06	0.04	0.16	0.09
Clostridium_IV	0.02	-0.20	0.05	-0.00	0.15	-0.21
Clostridium_sensu_stricto	0.48	-0.04	0.00	0.03	-0.00	-0.01
Clostridium_XlVa	0.18	-0.12	0.08	-0.07	-0.29	0.13
Clostridium_XVIII	0.06	0.27	-0.06	-0.14	-0.13	-0.18
Coprobacter	0.02	-0.21	0.14	0.17	-0.06	0.15
Coprococcus	0.19	0.07	-0.12	0.04	0.27	0.21
Dialister	-0.13	-0.12	0.05	0.35	0.25	0.22
Dorea	-0.02	-0.06	0.06	0.07	0.24	-0.02
Dysosmobacter	-0.31	-0.12	-0.20	0.06	-0.32	-0.20
Eggerthella	-0.08	0.01	-0.16	-0.03	-0.13	-0.21
Enterocloster	0.00	-0.14	0.02	-0.03	-0.33	-0.06
Enterococcus	0.15	0.10	-0.36	-0.04	-0.00	0.01
Faecalibacillus	0.27	0.03	0.04	0.06	0.29	0.19
Flavonifractor	0.00	-0.07	-0.02	-0.10	-0.34	-0.16
Frisingicoccus	-0.16	-0.25	0.05	-0.02	0.07	0.00
Fusobacterium	0.34	0.14	0.06	-0.15	-0.21	-0.03
Intestinimonas	0.10	-0.05	-0.02	-0.13	-0.20	0.08
Lachnospira	0.21	-0.11	0.05	0.03	0.24	0.12
Lacticaseibacillus	-0.19	0.07	-0.06	-0.09	-0.03	-0.20
Lactobacillus	-0.16	-0.06	-0.11	-0.27	-0.19	-0.26
Ligilactobacillus	-0.08	-0.07	0.05	-0.13	-0.38	-0.04
Limosilactobacillus	-0.07	-0.11	0.04	-0.21	-0.15	-0.29
Mediterraneibacter	-0.13	-0.41	0.17	0.23	0.02	0.16
Megasphaera	-0.12	-0.06	-0.42	0.04	-0.28	0.10
Negativibacillus	0.34	-0.21	-0.09	0.07	-0.27	-0.13
Neglecta	-0.46	0.08	-0.09	0.19	0.07	-0.01
Odoribacter	-0.12	-0.26	0.15	0.10	0.09	0.20
Olsenella	-0.22	-0.14	-0.00	-0.05	0.03	0.02
Oscillibacter	0.23	0.08	-0.12	0.04	0.29	0.10
Paraprevotella	-0.50	-0.36	-0.03	0.09	0.09	-0.21
Parasutterella	0.07	-0.22	-0.06	-0.03	-0.07	-0.15
Phascolarctobacterium	0.06	0.18	-0.05	-0.19	0.03	-0.26
Prevotella	-0.26	-0.32	-0.06	0.12	-0.04	-0.03
Prevotellamassilia	0.00	-0.09	-0.27	-0.01	-0.07	0.08
Romboutsia	0.24	0.17	0.29	0.17	0.09	0.07
Roseburia	0.17	0.09	0.08	-0.12	0.35	0.01
Ruminococcus	-0.25	-0.06	-0.05	-0.03	0.28	0.05
Ruminococcus2	-0.04	0.02	0.36	0.14	-0.36	-0.07
Ruthenibacterium	-0.04	-0.04	0.21	-0.15	-0.25	-0.03
Streptococcus	-0.44	-0.06	-0.26	-0.39	-0.34	-0.61
Turicibacter	0.08	-0.04	0.13	0.05	0.38	0.02
Veillonella	0.14	0.12	-0.19	-0.24	-0.18	-0.16

Age group and sex

Supplementary Figure S1. The values of ALDEx2 effect size obtained by comparing the gastritis disease-affected and disease-free Japanese control groups. The comparisons were performed considering age and sex. Negative values of effect size indicate taxa that are more abundant in the disease-affected group than in the control group, and positive values indicate taxa that are less abundant. The values of effect size less than −0.2 are indicated with pink, and the values greater than 0.2 are indicated with light blue.

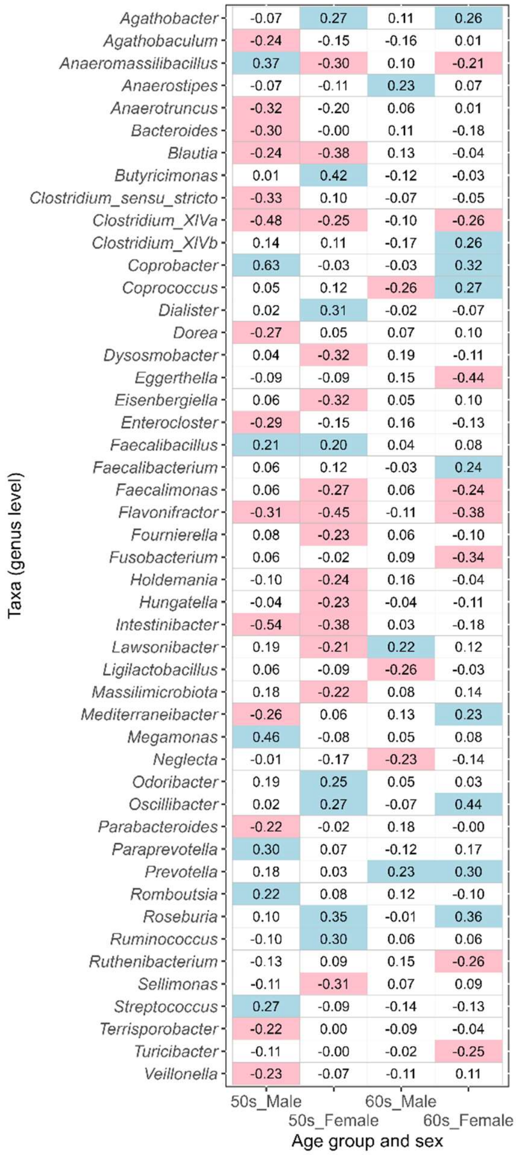

Supplementary Figure S2. The values of ALDEx2 effect size obtained by comparing the kidney disease-affected and disease-free Japanese control groups. The comparisons were performed considering age and sex. Negative values of effect size indicate taxa that are more abundant in the disease-affected group than in the control group, and positive values indicate taxa that are less abundant. The values of effect size less than −0.2 are indicated with pink, and the values greater than 0.2 are indicated with light blue.

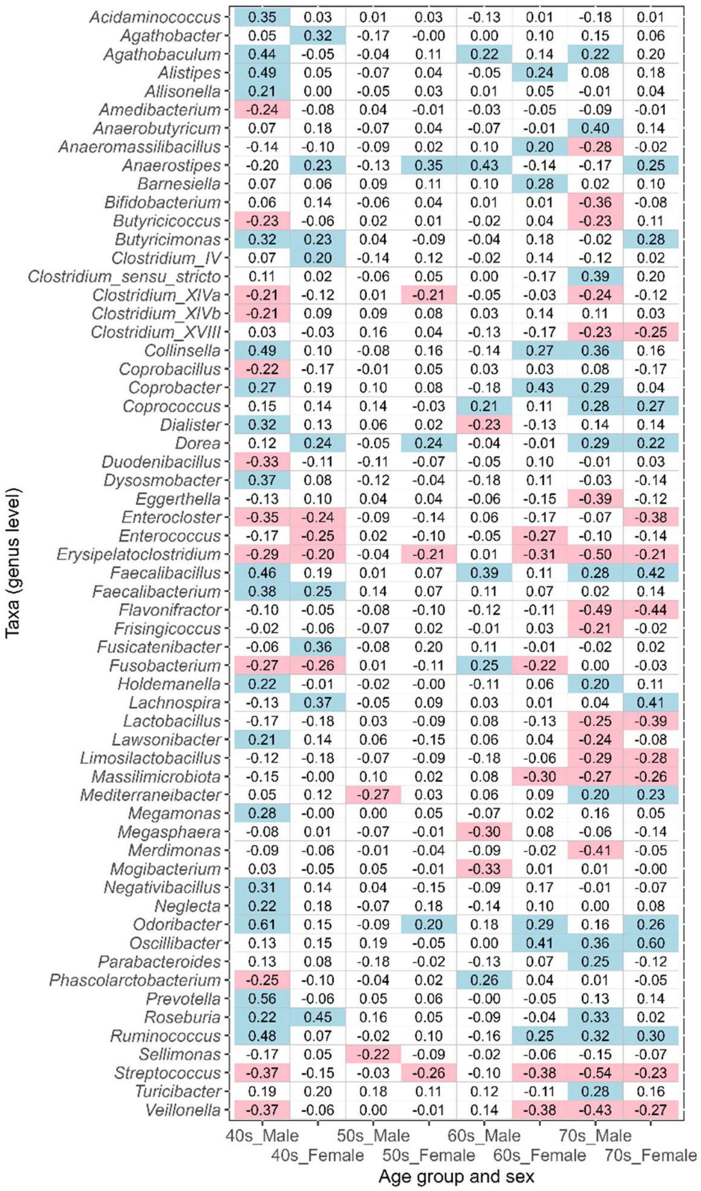

Supplementary Figure S3. The values of ALDEx2 effect size obtained by comparing the liver disease-affected and disease-free Japanese control groups. The comparisons were performed considering age and sex. Negative values of effect size indicate taxa that are more abundant in the disease-affected group than in the control group, and positive values indicate taxa that are less abundant. The values of effect size less than −0.2 are indicated with pink, and the values greater than 0.2 are indicated with light blue.

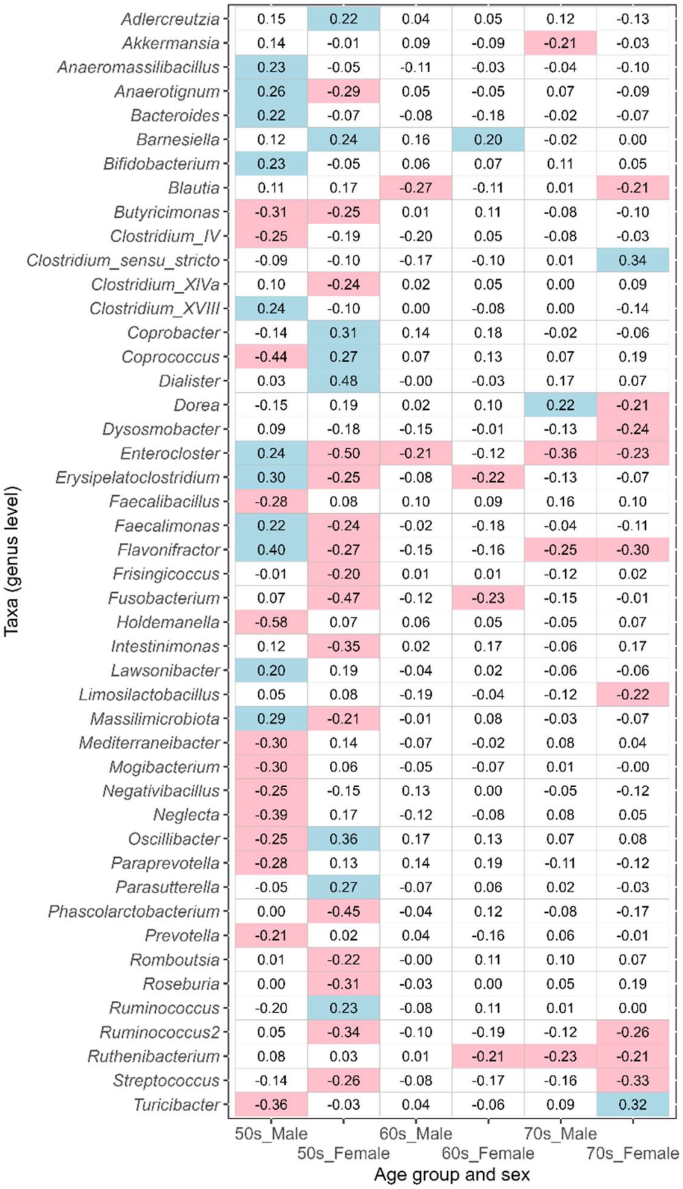

Supplementary Figure S4. The values of ALDEx2 effect size obtained by comparing the arrhythmia disease-affected and disease-free Japanese control groups. The comparisons were performed considering age and sex. Negative values of effect size indicate taxa that are more abundant in the disease-affected group than in the control group, and positive values indicate taxa that are less abundant. The values of effect size less than −0.2 are indicated with pink, and the values greater than 0.2 are indicated with light blue.

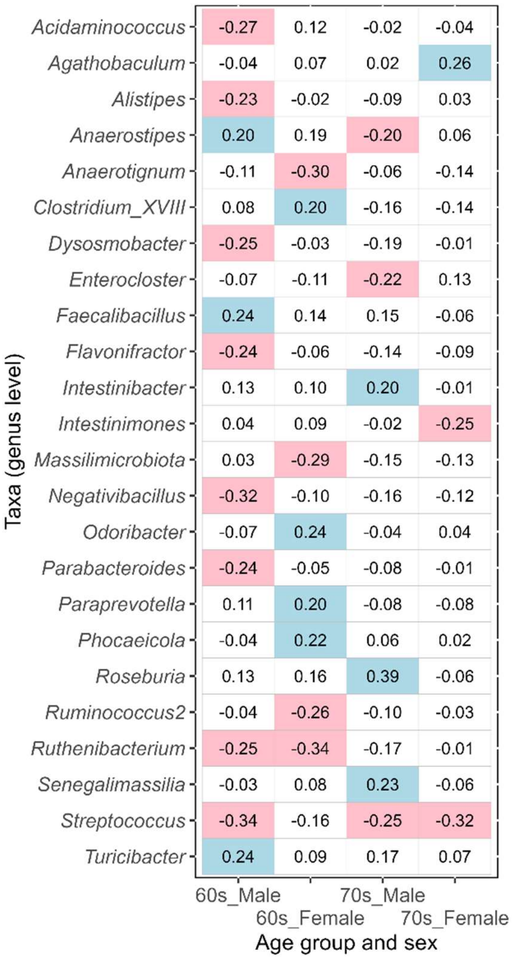

Supplementary Figure S5. The values of ALDEx2 effect size obtained by comparing the angina pectoris disease-affected and disease-free Japanese control groups. The comparisons were performed considering age and sex. Negative values of effect size indicate taxa that are more abundant in the disease-affected group than in the control group, and positive values indicate taxa that are less abundant. The values of effect size less than −0.2 are indicated with pink, and the values greater than 0.2 are indicated with light blue.

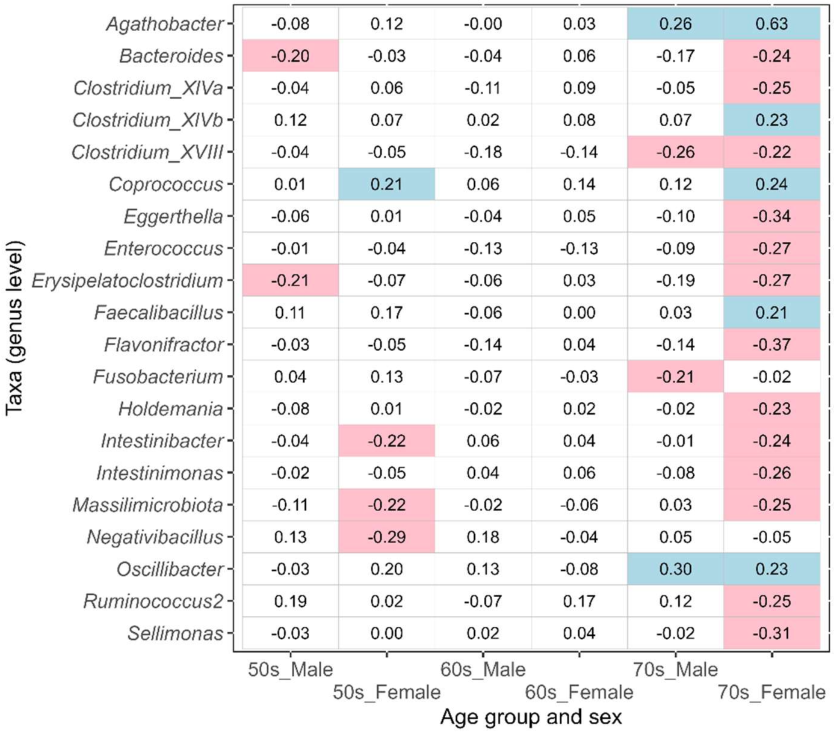

Supplementary Figure S6. The values of ALDEx2 effect size obtained by comparing the glaucoma disease-affected and disease-free Japanese control groups. The comparisons were performed considering age and sex. Negative values of effect size indicate taxa that are more abundant in the disease-affected group than in the control group, and positive values indicate taxa that are less abundant. The values of effect size less than −0.2 are indicated with pink, and the values greater than 0.2 are indicated with light blue.

Taxa (genus level)	20s_Male	20s_Female	30s_Male	30s_Female	40s_Male	40s_Female	50s_Male	50s_Female	60s_Male	60s_Female	70s_Male	70s_Female
Adlercreutzia	0.03	0.03	0.17	-0.01	-0.10	0.08	0.11	-0.02	0.05	-0.04	0.10	0.20
Agathobacter	0.34	0.08	0.00	0.01	-0.04	0.13	-0.10	0.15	-0.23	0.13	0.24	0.09
Agathobaculum	-0.00	-0.03	0.01	0.04	0.11	0.03	0.10	0.10	0.10	0.05	-0.00	0.25
Allisonella	0.06	0.03	0.07	-0.01	-0.01	0.03	-0.08	0.03	-0.25	0.07	0.18	0.08
Amedibacterium	-0.24	-0.04	0.09	0.01	0.06	-0.06	0.08	-0.00	-0.16	-0.02	0.02	0.02
Anaeromassilibacillus	0.06	-0.07	-0.04	0.04	-0.01	0.01	-0.16	-0.12	-0.09	-0.08	-0.24	-0.00
Anaerotignum	-0.05	0.08	-0.03	-0.03	0.06	0.01	0.10	-0.14	0.32	-0.08	-0.16	-0.13
Bacteroides	0.09	-0.11	-0.13	-0.01	0.02	0.05	-0.21	-0.10	-0.10	-0.29	0.15	-0.07
Bifidobacterium	-0.11	-0.13	0.04	0.16	-0.08	-0.01	-0.34	0.10	-0.06	-0.26	0.21	-0.03
Butyricimonas	-0.12	-0.07	-0.06	0.06	0.02	0.07	0.05	-0.07	0.07	0.31	-0.16	0.03
Clostridium_sensu_stricto	0.19	-0.19	-0.04	-0.01	0.04	0.06	0.02	-0.14	-0.23	-0.10	0.20	-0.07
Clostridium_XIVa	0.12	-0.06	-0.16	-0.17	0.02	-0.10	-0.26	-0.15	0.15	-0.23	-0.05	0.03
Clostridium_XVIII	-0.03	-0.01	-0.06	-0.09	0.11	-0.01	0.14	0.04	0.00	0.02	0.22	-0.12
Collinsella	0.14	-0.02	0.12	0.06	-0.07	0.04	-0.15	0.08	-0.05	-0.03	0.23	0.08
Coprobacter	-0.16	0.31	-0.14	0.01	0.06	0.03	0.15	-0.04	0.13	-0.02	0.12	0.10
Coprococcus	0.02	0.06	0.08	-0.07	-0.05	0.10	0.06	0.09	0.17	-0.00	0.11	0.30
Dialister	0.17	0.21	0.00	0.07	0.04	-0.00	0.05	0.05	0.25	0.15	-0.13	0.22
Dysosmobacter	0.02	0.01	-0.06	-0.02	0.01	0.01	-0.29	-0.08	0.15	-0.16	-0.33	-0.09
Eggerthella	0.23	-0.10	-0.15	-0.08	0.05	-0.09	-0.29	-0.09	-0.01	-0.15	0.16	-0.11
Eisenbergiella	0.06	-0.01	-0.08	-0.03	0.05	-0.05	-0.00	-0.06	0.10	-0.22	0.02	-0.07
Enterocloster	0.15	-0.08	-0.06	-0.16	-0.05	-0.07	-0.25	-0.18	0.04	-0.23	-0.27	-0.15
Enterococcus	-0.02	-0.02	-0.08	-0.04	0.02	-0.02	-0.05	-0.09	-0.01	-0.16	-0.25	0.05
Erysipelatoclostridium	0.01	-0.11	0.01	-0.20	0.02	-0.13	-0.16	-0.14	0.09	-0.17	0.10	-0.37
Eubacterium	-0.03	-0.07	-0.01	-0.03	-0.01	-0.04	0.02	-0.05	-0.08	-0.06	-0.12	-0.22
Faecalibacillus	-0.08	0.01	-0.09	-0.02	0.13	0.05	0.09	0.10	0.28	0.06	0.29	0.50
Flavonifractor	0.02	-0.06	-0.11	-0.08	0.02	-0.04	-0.37	-0.16	-0.03	-0.09	-0.42	-0.13
Fusobacterium	-0.08	0.02	-0.11	-0.03	0.06	-0.06	0.02	-0.07	0.25	0.04	0.11	-0.11
Holdemanella	-0.12	0.01	0.08	-0.01	0.05	0.04	0.16	0.03	0.20	-0.05	0.22	0.11
Intestinibacter	-0.03	-0.16	-0.07	-0.10	0.14	-0.06	-0.38	-0.06	0.02	-0.01	0.14	0.18
Intestinimonas	-0.27	0.10	-0.03	-0.01	0.02	0.01	0.13	-0.02	0.19	-0.13	-0.09	0.03
Lachnospira	-0.04	-0.03	0.06	0.00	0.08	0.03	0.14	0.03	-0.22	0.10	0.23	0.12
Lactobacillus	0.06	0.01	-0.06	-0.02	-0.00	-0.03	0.10	0.01	-0.00	-0.08	-0.34	-0.04
Lawsonibacter	-0.04	0.04	0.15	0.00	0.07	0.03	0.00	-0.04	0.00	0.17	-0.42	-0.08
Ligilactobacillus	-0.10	-0.05	-0.00	-0.01	-0.03	-0.03	-0.03	-0.03	-0.11	-0.00	-0.38	-0.01
Limosilactobacillus	-0.12	0.04	0.02	-0.00	-0.01	-0.02	0.03	-0.00	-0.03	0.09	-0.46	-0.25
Mediterraneibacter	0.07	-0.07	-0.01	-0.04	-0.03	-0.01	0.35	0.03	-0.24	0.09	0.28	0.28
Megamonas	-0.19	0.01	0.04	0.00	-0.04	0.06	0.06	0.08	-0.25	0.01	-0.16	0.08
Megasphaera	0.14	-0.10	-0.00	0.05	-0.07	-0.05	-0.23	0.01	-0.25	-0.08	-0.09	-0.10
Merdimonas	0.11	-0.17	-0.10	0.04	-0.04	-0.04	0.08	-0.10	-0.04	-0.16	-0.22	0.07
Negativibacillus	0.06	0.02	-0.06	0.01	-0.02	0.03	0.23	-0.07	0.38	-0.16	-0.18	-0.22
Odoribacter	-0.02	0.12	-0.17	-0.02	-0.01	0.03	0.04	-0.06	0.24	0.01	-0.04	0.34
Oscillibacter	0.08	0.08	0.10	0.11	-0.07	0.08	0.24	0.07	0.11	0.04	-0.04	0.09
Paraprevotella	0.03	0.03	0.02	0.02	0.01	-0.00	0.10	0.07	0.02	0.24	-0.07	-0.34
Phascolarctobacterium	-0.05	-0.21	0.01	0.03	0.06	0.05	0.08	-0.05	-0.09	-0.14	0.03	-0.40
Romboutsia	0.16	-0.05	-0.04	0.06	0.19	0.02	-0.05	0.12	-0.43	-0.13	0.14	-0.03
Roseburia	-0.15	0.03	0.01	0.08	0.06	0.03	0.08	0.20	-0.17	0.25	0.27	-0.15
Ruminococcus	0.13	0.08	0.03	0.06	0.11	-0.03	0.26	-0.01	0.19	0.08	0.08	0.00
Ruminococcus2	-0.08	0.04	-0.08	-0.01	0.14	-0.01	-0.18	-0.10	-0.06	-0.03	-0.32	0.33
Ruthenibacterium	0.02	-0.11	-0.11	-0.02	-0.13	-0.04	-0.19	-0.20	0.01	-0.22	-0.21	-0.05
Sellimonas	0.05	-0.01	-0.00	0.06	-0.01	-0.09	-0.20	-0.09	0.10	-0.17	-0.00	-0.09
Senegalimassilia	0.08	-0.02	-0.02	-0.00	-0.03	0.02	-0.13	0.05	0.02	-0.02	0.20	0.04
Slackia	-0.08	0.06	0.07	-0.01	-0.06	0.05	0.20	0.02	0.23	-0.03	0.14	-0.02
Streptococcus	-0.05	-0.07	-0.06	-0.04	-0.10	-0.04	-0.20	0.10	0.04	-0.02	-0.36	-0.12
Sutterella	-0.10	-0.07	0.05	0.05	0.09	0.02	0.05	0.13	0.14	-0.01	0.56	-0.11
Turicibacter	-0.02	-0.09	-0.05	0.09	0.09	0.10	0.03	0.09	-0.01	0.08	0.29	-0.16
Veillonella	-0.17	-0.13	-0.02	0.05	0.07	-0.01	-0.03	-0.06	-0.32	0.02	-0.21	-0.02
Victivallis	-0.01	0.02	0.07	0.03	-0.13	0.03	0.09	-0.06	0.09	-0.08	-0.29	0.13

Age group and sex

Supplementary Figure S7. The values of ALDEx2 effect size obtained by comparing the atopic dermatitis disease-affected and disease-free Japanese control groups. The comparisons were performed considering age and sex. Negative values of effect size indicate taxa that are more abundant in the disease-affected group than in the control group, and positive values indicate taxa that are less abundant. The values of effect size less than −0.2 are indicated with pink, and the values greater than 0.2 are indicated with light blue.
